# The Changing Role of Loop Diuretics in Heart Failure Management across the Last Century

**DOI:** 10.3390/jcm13061674

**Published:** 2024-03-14

**Authors:** Alberto Palazzuoli, Pietro Mazzeo, Martino Fortunato, Christian Cadeddu Dessalvi, Enrica Mariano, Andrea Salzano, Paolo Severino, Francesco Fedele

**Affiliations:** 1 Cardiovascular Diseases Unit Cardio Thoracic and Vascular Department, Le Scotte Hospital, University of Siena, 53100 Siena, Italy; 2 Cardiovascular Department, Azienda Ospedaliera Regionale “San Carlo”, 85100 Potenza, Italy; pietromazzeo10745@gmail.com; 3 Cardiology Division, San Pio Da Pietrelcina Hospital, Azienda Ospedaliera Regionale “San Carlo”, 66054 Marsicovetere, Italy; 4 Clinical Cardiology, AOU San Giovanni di Dio e Ruggi D’Aragona, 84131 Salerno, Italy; dottor.martinofortunato@gmail.com; 5 Department of Medical Sciences and Public Health, University of Cagliari, 09124 Cagliari, Italy; cadedduc@unica.it; 6 Department of Cardiology, Policlinico Tor Vergata, 00133 Rome, Italy; enrica@dottoressamariano.it; 7 Cardiac Unit, AORN A Cardarelli, 80131 Naples, Italy; andre.salzano@gmail.com; 8 Cardiac Unit, University Hospital of Leicester, Glenfield Hospital, Leicester LE3 9QP, UK; 9 Department of Clinical, Internal, Anesthesiology and Cardiovascular Sciences, Sapienza University Policlinico Umberto I, 00161 Rome, Italy; paoloseverino@uniroma1.it (P.S.); francescofedele@uniroma1.it (F.F.); 10 Istituto Nazionale Ricerche Cardiovascolari (INRC), 40126 Bologna, Italy

**Keywords:** heart failure, treatment, congestion, diuretics, diuretic resistance

## Abstract

Congestion is the main therapeutic target of acute heart failure (HF) treatment, and loop diuretics (LDs) are widely used drugs for this purpose. Despite their extensive use, these agents remain largely understudied in terms of modality administration, treatment duration, and escalation dose for subjects responding poorly to therapy. LDs were initially investigated in several edematous statuses such as cirrhosis, nephrotic syndrome, and congestive HF and initially approved for the treatment of cardiogenic congestion in 1966. Despite the long history and the undoubted role in congestion management, the use of LDs in the acute phase is mostly based on the physician’s experience, the oral amount chronically administered, and clinical decongestion response. Recent literature suggests monitoring diuretic activity by the evaluation of daily diuresis, weight loss, and sample urinary sodium assessment after early intravenous LD administration. More recently, the measurement of urinary sodium integrated with urinary and blood creatinine values and fluid status has been suggested as optimal marker to predict whole diuretic efficiency and to target the optimal dose. However, this method is not easily available in the chronic setting or in patients with recurrent hospitalization taking a high loop diuretic amount. Since high loop diuretic dose is related to diuretic resistance (DR) and poorer outcome, additional diuretics acting in different nephron sites are often required. Current sequential nephron blockade can stimulate diuresis by synergic mechanisms. This strategy is attempted in patients with poor response, revealing good results in the early period, but the effects of neuro-endocrine stimulation and electrolyte balance across long-term follow-up are still questioned. This paper reviews the historical course of loop diuretics and highlights the need for a universal approach based on clinical conditions, cardio–renal interactions, and HF phenotypes.

## 1. Introduction

Epidemiological data report congestion as the main cause for hospital admission in heart failure (HF) patients, representing a major therapeutic target in both acute and chronic settings. Indeed, the first step of the bed side clinical evaluation is based on congestion assessment and perfusion status and is mandatory for the beginning and the optimization of early treatment [1]. Notably, loop diuretic (LD) treatment remains the most common approach, used in at least 90% of cases, and the main therapeutic weapon to address fluid accumulation and overload [2,3]. This is due to the capacity of LDs to improve HF symptoms and reduce central and peripheral edema [4,5]. They are strongly recommended by guidelines, representing the cornerstone to improving symptoms and signs of congestion (class I, level of evidence C) regardless of phenotype and setting. On the other hand, loop diuretic use has been associated with an increase rate of mortality and hospitalization, particularly when they are administered in higher doses and through a continuous infusion [6,7]. Therefore, no clear evidence about the correct use of LDs exists, and the correlation between LD amount and poor outcome deserves a detailed analysis considering HF severity, association with renal dysfunction, and co-morbidity burden [7,8]. Classically, the correct use and the proper administration dose during acute hospitalization and pre-discharge are determined based on a single physician’s experience, common center habit, and the oral dose administered before hospitalization. In this context, a recent statement from the HF association of the European Society of Cardiology (HFA-ESC) provided some recommendations on the correct use of diuretics based on diuresis extent, urinary sodium excretion, and response to the initial dose [9]. Despite these suggestions, many concerns related to the use of loop diuretics, the administration modality, timing of infusion therapy, and shifting to oral administration remain unanswered. Few randomized controlled studies have evaluated all these features in detail, and in the last century some protocols have been attempted to identify the correct diuretic use [10,11,12]. To date, a precise algorithm of treatment is still lacking [13,14]. In this paper, we review the application of diuretics in the last five decades, and we purpose a strategy based on previous reports to recognize an optimized therapy based on clinical and hemodynamic patient profiles. 

## 2. Loop Diuretic Administration and Application during the Last Century

The first loop diuretics (specifically, ethacrynic acid and furosemide) were developed in the first years of the 1950s and released about ten years later, leading to their prescription in several diseases characterized by edema, such as heart failure, nephrotic syndrome, and liver failure/cirrhosis [15,16,17]. While relieving congestion, these drugs can also lead to a worsening of the renal function, from a slight decrease in the glomerular filtration rate to end-stage renal disease.

They are habitually used to rapidly alleviate symptoms of fluid overload and congestion. However, despite their use for about 60 years, international guideline recommendations still remain mostly based on expert opinion and general habits [18,19]. Intravenous loop diuretics still represent the mainstay of HF treatment and are administered to approximately 90% of hospitalized patients. However, decades of clinical experience with these agents and data from the literature cannot shed light on the best administration modality or more adequate dosage. Consequently, both the mode of administration and dose used in clinical practice widely vary around the world [20,21,22].

The LDs available in the last sixty years consist of furosemide, torasemide, bumetanide, and ethacrynic acid (with the latter as the only lacking the sulfonamide moiety). 

Mechanistically, they provide an inhibition of the Na^+^/K^+^/2Cl^−^ co-transporters that are present at level of the thick ascending limb of the loop of Henle, accounting for reabsorption of 20% to 30% of the total filtered sodium. As a result, LDs inhibit reuptake of sodium and water and increase urinary chloride, calcium, and magnesium excretion. 

Despite being the first FDA approved loop diuretic in 1966 and the most frequently used, there are limited data supporting the superiority of furosemide when compared to other available LDs. Furosemide has a widely ranging bioavailability (10–100%), and different inter- and intra-individual properties are associated with the wide variation for urinary excretion of furosemide (25–42%). On the other hand, torasemide and bumetanide display a more consistent bioavailability that ranges consistently from 80 to 100%, and torasemide has the longest half-life of the loop diuretics (3–4 h) [23,24,25,26]. 

In addition, Torasemide, approved by the FDA in 1993, appears to have potential favorable effects on the renin angiotensin aldosterone system mineralocorticoid antagonizing properties and antifibrotic effects on myocardium [27]. The Torasemide in Congestive Heart Failure (TORIC) study was an open-label prospective cohort involving 1377 patients with NYHA class II–III, comparing oral torasemide to furosemide [28]. The study confirmed torasemide safety and tolerability, showing a major improvement in NYHA functional class and a lower rate of hypokalemia; finally, even if not designed to investigate mortality, patients attributed to torasemide arm displayed a lower mortality during follow-up. However, the subsequent TRANSFORM-HF trial, involving 2859 participants hospitalized with heart failure (regardless of phenotypes), did not show any significant differences in main outcome (i.e., all-cause mortality) over the follow-up (12 months) [29]. Finally, a metanalysis showed a significant difference between furosemide and torasemide in the increase in the urine volume and on the effect on potassium excretion. In addition, despite no significant differences between torasemide and furosemide shown in mortality, weight loss, edema improvement, heart rate, or systolic/diastolic blood pressure, torasemide showed a superior effect in improving ejection fraction and a significantly lower hospital stay [30].

Ethacrynic acid is the oldest loop diuretic developed, together with furosemide; however, the two compounds are chemically dissimilar. Ethacrynic acid is an α → β unsaturated ketone derived from phenoxyacetic acid; conversely, furosemide is an anthranilic acid derivative which has in common with thiazides a sulfanyl group adjacent to a chloride on the benzene ring [31,32,33]. As furosemide, ethacrynic acid is the strongest diuretic currently available, acting by the inhibition of Na reabsorption along the ascending loop of Henle, with a possible site of action in the proximal tubular [34,35]. Similar to furosemide, ethacrynic acid has a rapid and short action with steep dose–response curves; as a result, it can be used regardless of the presence of hypoalbuminemia or electrolyte or acid-base imbalance. Xipamide is a diuretic derived from salicylic acid and has a molecular similitude to chlorthalidone. Its pharmacodynamic profile shows a diuretic efficacy similar to that of frusemide (furosemide) at doses up to 40 mg, but the onset and duration of action are comparable to those of hydrochlorothiazide. Xipamide has been studied mostly in the treatment of mild to moderate essential hypertension, with few controlled studies in edematous states, suggesting that xipamide 40 to 80 mg is comparable in efficacy to equal doses of frusemide and that the side effects of hypokalemia, hyperuricemia, and increased blood glucose in patients with diabetes or latent diabetes are similar to those of other diuretics.

### Route of Administration

LDs can be administered both orally and intravenously. When given intravenously as bolus injections, vigorous and rapid diuresis is stimulated. However, several worries regarding this route of administration have been raised: Giving intermittent boluses may lead to marked fluctuations in intravascular volume, thus increasing their toxicity; on the other hand, the use of repetitive, large doses may develop an acute tolerance to the drug. On the other hand, it was proposed that continuous infusion reduces the intravascular volume fluctuation, leading to a relatively constant urine output. To date, few randomized controlled trials have compared the two different methods of administration (i.e., bolus intravenous administration vs. continuous infusion), with conflicting results [36,37,38,39,40,41,42,43,44] (see Table 1). 

The main randomized controlled studies were published from 1992 to 2004 [46]. All the studies differ with regard to dosage, duration of infusion, observation period, the number of patients involved, and the total follow-up. Specifically, the low- to moderate-dose studies investigated furosemide in a range of 80 to 320 mg/24 h, while torasemide was used at a dose of 100 mg/24 h. The duration of infusion varied widely, ranging from 30 min to 48 h. The observational period was limited to the hospitalization period ranging from 24 h to 12 days. The largest study involved 107 patients with longer follow-up, while the remaining studies involved few patients. Again, the follow-up period was very different, ranging from 24 h to 48 h with only one study (also having morbidity and mortality as endpoints) lasting up to 31 months. The larger comprehensive clinical trial was performed in 2011, the Diuretic Optimization Strategies Evaluation (DOSE) trial. It was a randomized protocol sponsored by the NHLBI Heart Failure Clinical Research Network that aimed to compare continuous infusion of IV furosemide vs. bolus, as well as low-dose vs. higher-dose therapy. Even if this study found no difference in the primary endpoint, the higher-dose diuretics regimen (i.e., 2.5 time more than home dosage, at least 80 mg) was more effective (in term of greater change in weight, net fluid loss, and relief of dyspnea) than the lower-dose regimen (i.e., same dosage as at home), without clinically important negative effects on renal function [47].

In the Diur-HF Trial, despite a significant reduction in BNP levels, a higher rate of adverse events in patients developing novel renal impairment during hospitalization was shown; however, the mean diuretic regimen was higher in this study (80 ± 20 mg with progressive increase from 160 ± 40 to 250 ± 40 mg) when compared to the DOSE trial [48,49]. 

In conclusion, by the present analysis of the literature, it is not possible to outline a better modality of loop diuretics administration as well as dosage, since most analyses were limited to rough parameters like weight loss, diuresis, and symptom score measurement. Additionally, most findings are extracted from retrospective studies. Finally, the current issues deserve a more detailed investigation by a specific well-designed prospective trial comparing different administration strategies, dose infusion, amount, and related effects on hard endpoints.

## 3. Loop Diuretic Treatment in Acute Heart Failure

A natural history of heart failure is characterized by fluid accumulation and recurrent episodes of decompensation, mainly related to fluid accumulation and retention—i.e., acute heart failure [50,51]. 

Congestion, related to pressure and/or volume overload, is a crucial feature influencing pathophysiology, severity, and prognosis of HF irrespective of HF phenotypes (e.g., HFrEF vs. HFpEF) [3,4]. Despite a few patients with acute heart failure presenting with signs and symptoms of low perfusion, signs and symptoms of congestion are the main reasons of hospitalization and urgent medical care in HF patients [39,40]. In this framework, diuretics represent a cornerstone of HF therapy with guidelines strongly recommending the use of LDs as first line agents to alleviate fluid overload signs and symptoms (class I, level of evidence C) [14,52]. However, before starting LD treatment, it is necessary to investigate whether volume overload or volume redistribution is causing the congestion [3].

The main objectives in the therapy of patients with acute congestion and volume overload are as follows: i. decongestion without residuary volume overload; ii. adequate perfusion pressures to maintain a proper organ perfusion; iii. maintenance of guideline-directed medical therapies that can increase the diuretic response and improve long-term survival [53,54]. Patients with decompensated HFrEF or HFpEF can present a similar congestion profile [54], and the goal of diuretic therapy is similar throughout the HF spectrum. Once euvolemia has been reached, LD therapy should be reduced to the lowest dose able to maintain optimal fluid balance [54,55]. Loop diuretics require adequate plasma concentration and renal perfusion, which is often reduced in patients with heart failure [56].

In patients undergoing chronic loop diuretic therapy, LDs induce, through hypertrophy of tubular cells, compensatory distal tubular sodium resorption; as a result, the natriuresis is reduced [14]. Guideline recommendations are in favor of the use of intravenous LDs in AHF, as oral diuretic uptake (furosemide in particular) can be reduced because of congestion due to bowel edema (class I, level of evidence C) [14,57]. This is consistent with wide bioavailability of orally administered furosemide (10–90%) [8]. The oral bioavailability for bumetanide and torasemide is about 80–90%, with torasemide showing a longer half-life [16]. A threshold of concentration, in order to invoke natriuresis, is exhibited by LDs; therefore, a small drug dose is necessary prior to exceeding the baseline rate of sodium excretion [58,59]. Thereafter, a linear increase in dose is required to reach a maximum in the natriuresis. Despite an additional increase in the LD dose, this maximum does not result in a higher peak of natriuretic response; it leads to a longer period in which LDs are over the threshold level, resulting in an increase in the total natriuresis. Likewise, multiple administrations can cause additional natriuresis because of the increase in the duration of time above the natriuretic threshold. Guidelines recommend that patients naïve to LDs should receive at least a 20–40 mg furosemide equivalent as a starting dose, with a higher dose only in patients with pre-existing kidney dysfunction [9,60]. On the other hand, patients already receiving an oral dose should receive at least the same oral dose administered intravenously. Because high doses may lead to neurohormonal activation and electrolyte imbalance [8,61,62], guidelines suggest starting with low doses to evaluate the diuretic response and eventually intensify the dosage if insufficient. Furosemide can be administered as fractionated daily boluses (e.g., 2 or 3) as well as a continuous infusion. Due to the possibility of a post-dosing sodium retention, a single bolus administration is not recommended [9,45,60]. When a continuous infusion regimen is chosen, a loading dose may be used to accelerate the attainment of the steady state. If bolus infusion is preferred, doses should be administered at least with a 6 h interval, with the aim of maximizing the time above the natriuretic threshold and of avoiding the described rebound sodium retention. The CLOROTIC trial, a prospective, double-blind, placebo-controlled trial, shows that the addition of idroclorotiazide to loop diuretic therapy improved diuretic response in patients with AHF [63]. The results of this study are consistent with those of the recent ADVOR trial, which found the addition of acetazolamide to intravenous loop diuretic therapy in patients with AHF resulted in a greater incidence of successful decongestion, despite a trend towards increased combined endpoint of mortality, and hospitalization was found in the active arm [64].

In routine clinical practice, diuretic response assessment is crucial; however, clinical signs of decongestion, the amount of diuresis and weight loss, and the evaluation of renal function display a limited sensitivity [65]. Direct urinary sodium monitoring represents a simple indicator of diuretic response; however, the cumulative 24 h sodium output measurement may be challenging. It has been suggested that a post-diuretic spot urine sodium ranging from 50 to 70 mmol/L is associated with the worst outcome (i.e., worsening HF, worsening kidney function, and long-term adverse events) [66,67,68,69]. A urine sodium amount >50–70 mEq/L at 2 h or a urine output >100–150 mL/h during the first 6 h is considered a satisfactory diuretic response [9,69]. Even if this approach seems promising, overcoming several practical issues caused by the cumulative urine collection, further validation is necessary in larger populations [70].

**Figure 1:**
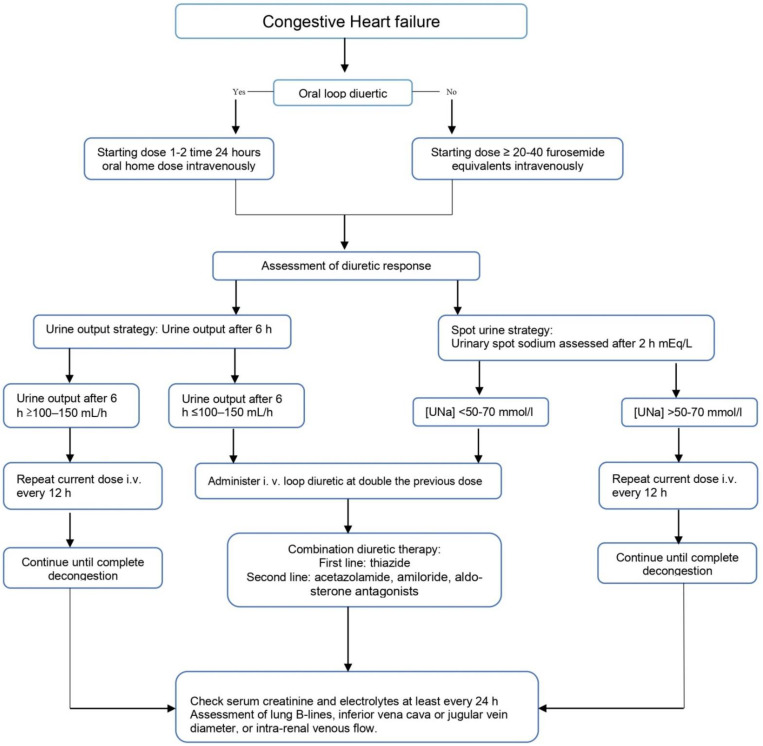
Flow chart describing a correct diuretic use and administration in acute HF starting from oral diuretic administration up to stepwise intravenous management. Extracted and modified from [9].

If diuretic response is poor, the loop diuretic dose can be doubled [9]. In addition, if inadequate diuretic response is proven, other diuretics (e.g., thiazides, metolazone, acetazolamide) in addition to LDs may be considered (see Figure 1), with a careful monitoring of renal function and serum electrolytes [9,71,72]. Loop diuretic dose should be progressively decreased at the achievement of a significant negative fluid balance [9,18]. In addition, the use of ultrasound, including serial assessment of lung B-lines, jugular vein diameter, inferior vena cava, or intra-renal venous flow, showed a potential utility in decongestion monitoring [73]. When the clinical condition is stable, oral treatment should be started and continued at the lowest dose able to prevent congestion [61]. Before discharge, decongestion should be completed, incomplete decongestion being a major predictor of poor outcome [74,75]. Finally, patients should be referred to a multi-disciplinary HF management program with the aim of promoting adherence and up-titration of disease-modifying therapy, performing timely follow-up, and screening for additive device-based intervention therapies, essential features for reaching an optimized treatment [14].

## 4. The Use of Loop Diuretic in Chronic Heart Failure

Diuretics are recommended in chronic heart failure with a class I level of evidence C to treat signs and symptoms of congestion, to improve HF symptoms and exercise capacity, and to reduce HF hospitalizations, regardless of ejection fraction [14]. However, no convincing data exist demonstrating a significant prognostic role of loop diuretic administration, particularly when high dosages are used [75,76] When using diuretics in the chronic setting, residual clinical congestion, especially in patients recently hospitalized for congestive heart failure, should be routinary evaluated [65]. Some clinical elements that can help to evaluate residual congestion are the relief of dyspnea (NYHA class), the jugular venous pulsation, and hepato-jugular reflux; the presence of hepatomegaly; the progression of edema; and the 6 min walk test. Other technical evaluations that can better recognize the exact congestion status are NT-proBNP/BNP dosage, chest X-ray, vena cava imaging, and lung ultrasound [77]. There are no large, prospective, randomized controlled trials investigating the association of LDs with morbidity and mortality in CHF patients, and the quality of the evidence regarding diuretics is poor; however, the major disease-modifying treatment trials were all conducted with patients assuming a high background loop diuretic therapy [8,14]. Several trials reported the positive effect of diuretics on mortality and worsening heart failure. Three placebo-controlled trials reported the effect of diuretic therapy on mortality in participants with heart failure with a lower mortality for subjects treated with LDs [78,79,80].

Specifically, an absolute risk reduction of 8% in mortality was observed [78,79,80]: De Jong et al. showed that patients who had withdrawn from diuretics had rebound edema [81]. Two parallel RCTs reported the effect of diuretics versus active control on exercise capacity, resulting in an improvement of exercise capacity [82,83].

Verel et al. observed that oral furosemide increases quality of life [84]. LD maintenance therapy, at the lowest dose necessary, should be kept with the aim of preventing congestion [85,86]. Importantly, individual diuretic requirements change significantly over time; notably, doubts still exist about the optimal post-discharge LD dose. Indeed, in patients developing AHF despite a previous LD treatment, a higher dose may be needed after discharge. Furthermore, switching to a different LD (e.g., from furosemide to bumetanide or torasemide) could be considered because of the more predictable pattern of absorption and bioavailability. However, a careful follow-up, especially early in the post-discharge period, is required to define the most appropriate outpatient diuretic dose. Therefore, since HF is a changing disease with different trajectories and fluctuation mainly due to treatment response, the use of loop diuretics may be routinely revised according to the hemodynamic clinical and neurohormonal response [9]. Further research is needed to evaluate ambulatory parameters of volume status, which may allow easier adjustment of LD treatment [87]. Currently, the use of additional categories of drugs with diuretic effects, such as sodium glucose co-transporter 2 inhibitors (SGLT2is), could lead to a change in the dosage of diuretics. A reassessment of the need for LDs after the start of a treatment able to improve cardiac status may lead to a change in or discontinuation of diuretic dosage [88,89]. A self-training evaluation and educational program addressing auto-evaluation based on assessment of the onset of new symptoms/signs of congestion and continuous weight assessment should be encouraged to avoid frequent hospitalization by a correct diuretic dose [59,64]. A description of each type of diuretic, the standard dose, and practical guidance on the use in chronic heart failure is shown in Table 2.

## 5. How to Evaluate Diuretic Response and Euvolemic Status

**Figure 2:**
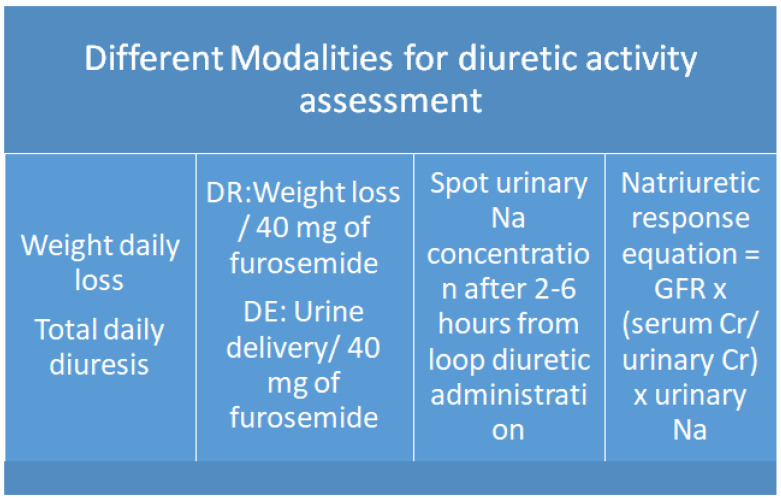
Mode of evaluation of diuretic activity and efficiency during intravenous administration.

Congestion status is usually assessed by clinical evaluation, although it is an unprecise approach which does not distinguish between intravascular and extravascular fluid retention or cardiac versus systemic congestion [90]. During diuretic treatment, it is mandatory to identify the correct diuretic amount needed in order to avoid recurrent congestion but also dehydration and electrolyte disorders. Accordingly, a correct balance between fluid intake and drug amount is one of the main targets in HF management. This status is arduous to achieve since the diuretic response is often subjective, depending on the contemporary presence of chronic kidney disease, the interstitial liquid mass, tubular resorption, intrinsic fluid overload fluctuation, right ventricular function, and central venous pressure [91]. Of note, a universal diuretic threshold capable of maintaining euvolemia in all HF subjects is a chimeric goal, and physicians may evaluate the balance between fluid intake and output with the aim of maintaining a negative status to avoid the physiologic fluid retention related to neuro-endocrine overdrive and cardio–renal crosstalk deterioration [18,92]. Behind common methods (diuresis and weight check), more precise methods have been proposed to better evaluate the diuretic response, commonly defined as weight loss gain per 40 mg of administered furosemide. This formula has been efficaciously tested in the PROTECT and RELAX trials, and it demonstrated a significant correlation with congestion score and outcome: patients with low values experienced a worse prognosis and have increased residual congestion at discharge [93]. Contemporarily, Testani et al. suggested an alternative modality in order to screen patients with efficacious vs. inefficient diuresis, matching 40 mg of furosemide per urine output [94]. Despite both approaches demonstrating a significant predictive power, they keep some weaknesses related to modest correlation with renal function and renal blood flow; in addition, they do not take into account the sodium and liquid balance and net liquid input–output measurement and initial dry or congested status. Accordingly, they cannot be applied in all HF patients, and some differentiation should be considered during evaluation of acute destabilized vs. chronic stable patients. Similarly, diuretic response may have reduced significance in the setting of hypovolemia and in diuretic-naïve subjects. Finally, both formulations cannot identify the correct diuretic regimen, particularly in acute decompensated setting, in which inter-patient variability in sodium content and diuretic-induced urine response exists [95]. Another feature to be accounted for in more advanced HF stages is the breaking phenomena, in which neuro-endocrine overdrive and renal hemodynamic alterations (in terms of increased renal vein pressure and arterial underfilling, impaired glomerular and tubular function, and hypochloremic alkalosis) are the main drivers of diuretic response [96]. Measurement of natriuresis could overcome many of the above cited limitations related to inaccuracy of exact fluid balance and lack of capture of extracellular volume expansion. Of note, the use of urine sodium concentration measurement after initial diuretic administration (1 or 2 h) showed relevant evidence for extensive application in clinical practice: low Na urine spot concentration below 50 mmol is associated with poor diuretic response, impaired decongestion during treatment, and reduced long-term survival [69,97]. Additionally, a positive sodium balance is associated with increased risk of poor outcome (death and hospitalization) even in subjects with negative fluid balance. Therefore, spot urinary sodium is highly related to 24 h Na collection, and it is predictive of tubular resistance and avidity. However, even this approach has some limitations due to the absence of diuretic dose and urine flow rate account measurements; moreover, individual variability related to pharmacodynamic properties such as blood drug concentration and renal diuretic curve may significantly change urine Na values [98]. Notably, subjects taking LDs as starting dose demonstrate minimal probability of developing diuretic resistance and high Na resorption in early administration compared with patients taking chronic loop diuretics in which reduced Na excreted may depend on salt intake and reduced proximal tubule diuretic secretion and decreased GFR. In the setting of chronic kidney diseases (CKDs), the renal blood flow redistribution and tubulo-glomerular feedback cause impaired diuretic response by the competition with organic anions and urate [99] (Figure 2). To date, a more comprehensive strategy including all these items in a single formula appears to be the best method to achieve diuretic response. Notably, the natriuretic response prediction equation based on spot urine Na and creatinine value obtained after 2 h from diuretic administration is capable of estimating urine production and the ratio of serum to creatinine together with Na excretion [70]. Overall, current applications suggest that physicians have become much more aware of some simple and available methods for diuresis prediction and response that could help in targeting diuretic dose and identifying the optimized loop diuretic amount based on underlying causes of diuretic resistance, pharmacological response, and intrinsic renal dysfunction.

## 6. How to Avoid Diuretic Resistance by Sequential Nephron Blockade

To achieve a natriuretic response, a sufficiently high concentration of an LD in tubular fluids is needed to block the Na^+^/K^+^/2Cl^−^ cotransporter. After a long period, LD administration leads to an increased sodium release to the distal tubular system, which might result in compensatory hyperplasia of tubular cells with the consequence of diuresis volume decline [100]. The altered medullary signal, due to increased peritubular oncotic pressure and intrarenal pressure promoting renal vasoconstriction and total blood flow decrease, causes an increased Cl-Na exchange with a consequent reduction in Na delivery. These disorders are the main pathophysiological processes of diuretic resistance. This condition may be defined as the inability to produce adequate diuresis/natriuresis despite a pertinent diuretic regimen [54]. It is revealed by enhanced Na avidity and increased tubular resorption. The addition of alternative diuretics with different tubular targets provides a synergic diuretic effect evading rebound sodium retention [71,96]. 

The first step of this approach to increase diuresis and to reduce diuretic resistance is to add a thiazide or thiazide-like diuretic to reduce Na reuptake in the distal tubule. Distal tubule diuretics affect the adaptive process of cell hyperplasia appearing after a long-term LD treatment [101].

Metolazone and hydrochlorothiazide are the most frequently used thiazides. Metolazone has been proposed as the first choice over other thiazide diuretics due to its supplementary effect exerted in the proximal tubule and its low cost [101,102]. However, there are no clear data evidencing its clinical benefit over other thiazide diuretics in terms of increasing net urine output, preservation of renal function, or preventing electrolyte impairment [103,104]. Recently, metolazone and intravenous chlorothiazide have been compared in a retrospective cohort study in patients with acute decompensated HF and evidence of loop diuretic resistance. In this population, chlorothiazide did not show superiority to metolazone in the 24 h urine output, and metolazone use was suggested as a potential cost-saving strategy [103,105].

Some authors, with the aim of determining a full blockade of the distal nephron, suggested administering thiazides before intravenous LDs; in this way, thiazides act before the distal nephron is engulfed with Na from the thick ascending limb. However, when oral loop diuretics are administered, this alternated dosing is not needed [106].

Although combination therapy has many advantages, it is also associated with a significant increase in adverse effects. One of its advantages is related to the ability to minimize hypocalcemia related to loop diuretics, given the action of thiazide diuretics on calcium metabolism. Moreover, thiazide diuretics loop diuretics effects when intermittently administered, this mechanism prolongs natriuresis and diuresis [107]. Additionally, adding a thiazide diuretic to loop diuretic therapy is beneficial in patients with a severely impaired eGFR, in which case the fractional Na excretion should be maximally increased to guarantee adequate natriuresis [19,71]. On the other hand, combination therapy also displays a significant increase in adverse events and requires careful monitoring. One of the drawbacks of thiazide diuretics is the limitation of the kidneys’ ability to dilute urine, reducing free water clearance; hence, they are not suitable in hypotonic hyponatremic statea. Other side effects are hypokalemia and dehydration [108]. Finally, some intriguing data come from the Cardiorenal Rescue Study in Acute Decompensated Heart Failure (CARRESS-HF), indicating that the use of thiazide-based therapy in the context of a diuretic efficacy-guided approach, when compared to the aggressive ultrafiltration approach, is able to enhance diuresis without impairing eGFR [109].

**Figure 3:**
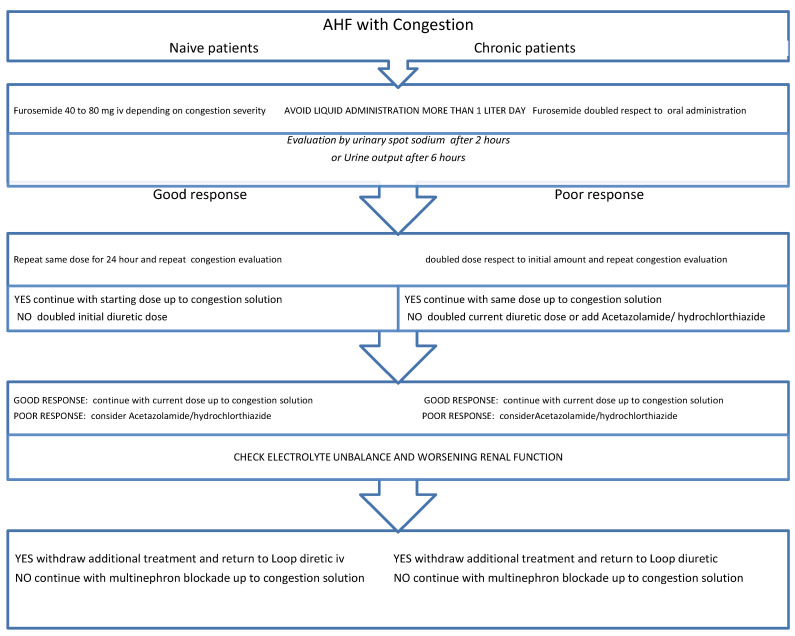
An alternative algorithm proposed for patients admitted with acute heart failure (AHF) and congestion. The flow chart includes naïve and chronic users of loop diuretics. Additionally, it includes a second step option the introduction of additional diuretics in those with poor natriuretic and diuretic response.

An old and mostly forgotten diuretic is acetazolamide, widely used in the past given its ability to boost LD efficacy. Acetazolamide prevents proximal Na reabsorption, producing higher Na concentrations, specifically in the loop of Henle. In addition, acetazolamide enhances thiazide-type diuretic efficacy and downregulates pendrin expression in the distal nephron [110]. Pendrin (the sodium-independent chloride/iodide transporter) can counterweigh for Na and Cl loss in the distal convoluted tubules and could be an unrecognized cause of resistance to LDs [111]. However, while acetazolamide displays a modest diuretic power, it represents a very effective booster of other diuretics efficacy when used in association [112]. This aspect is additionally sustained by a randomized trial including 24 congested patients refractory to loop diuretic therapy [113]. A larger randomized trial (ADVOR) has recently confirmed that the addition of acetazolamide is able to overcome a greatly reduced fractional sodium excretion, improving congestion signs and natriuretic peptide levels [64]. However, no data are available on the benefits of addition of acetazolamide, and the drug use should be limited to a short-term treatment given the increased risk of metabolic acidosis. Similarly, the CLOROTIC trials revealed a reduced congestion in patients taking hydrochlorothiazide without inducing electrolyte disorders or worsening renal function. Current positive findings achieved during acute phase, need to be confirmed across a longer follow-up period. Indeed, safety data of multiple diuretic combinations in post discharge and chronic phases are still lacking. Interestingly, if beneficial effects beyond decongestional properties will be confirmed in a longer period, the current diuretic administration algorithm could be modified according to the proposed protocols. This sequential approach may be introduced and integrated with respect to the Mullens position paper based exclusively on LD escalation dose. The use of different diuretics could be endorsed in patients showing poor response as reported in Figure 3.

High doses of mineralocorticoid receptor antagonists (MRAs: spironolactone, eplerenone, and finerenone) are recommended in HF due to their well-proven effects on hard outcomes (i.e., mortality and HF hospitalizations). Guidelines recommend their use in acute decompensated HFrEF, and they should be added to HF therapy of naïve patients. Although they are currently used in low doses, better results have been found when MRAs are increased to the target dose. However, whether there is a synergistic diuretic effect between MRAs and LDs it is not well established. In addition to reducing K wasting caused by LDs, MRAs show a mild but effective natriuretic effect [114]. Even if the rationale for MRA use may not rely on the sequential nephron blockade, MRAs are indicated in the long-term benefit when eGFR is stable and K levels are into the range [115,116,117]. Two recent trials demonstrated that finerenone in patients with chronic kidney disease (CKD), diabetes, and evidence of microalbuminuria is associated with reduced cardio–renal outcome. These findings are mainly driven by the reduction in HF occurrence and hospitalization. Additionally, the beneficial effect is similar in patients with a history of HF and in subjects without symptomatic HF [118,119].

**Figure 4:**
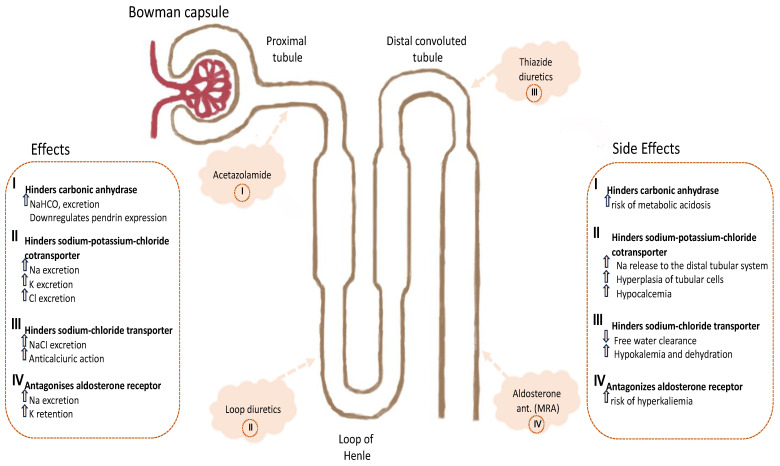
Different diuretic site actions and potential synergic mechanisms that could improve the loop diuretics effects when administered in association. The concomitant use may amplify common side effects such as electrolyte imbalance and permanent worsening renal function.

Finally, a new emerging agent demonstrating positive effects during hospitalization when associated with traditional diuretic treatment is the sodium–glucose cotransporter inhibitor (SGLT2i). In the EMPULSE study, the addition of empagliflozin to traditional decongestional treatment was associated with better quality of life exercise tolerance and reduced physical limitations [120]. This issue was associated with reduced markers of congestion weight loss and lower loop diuretic dose before discharge [121]. Additionally, the positive trend was observed in both de novo and acute decompensated patients regardless of ejection fraction or the presence of diabetes, and it was subsequently maintained for 3 months (Figure 4) [122].

## 7. Conclusions

Despite loop diuretics being the most common agents employed to avoid congestion in acute and chronic HF, structured randomized clinical trials directly evaluating the power and potential side effects of these drugs are still lacking. In addition, solid evidence on how to use loop diuretics, the modality of administration, and the correct dose escalation need to be better elucidated. Notably, current recommendations provide only general information on LD management during congestion phases, and a structured protocol based on solid findings is required. The evaluation of urinary sodium and diuresis during early administration appears to be the best modality to define the diuretic amount, although studies specifically addressing this issue are yet to be published. Similarly, in the chronic setting, no reports investigating the tailored loop diuretic dose according to underlying conditions, the modality to avoid diuretic resistance, or the long-term effect of sequential nephron blockade are available. Future studies specifically investigating all these issues and assessing the proper role of loop diuretics are needed.

## Figures and Tables

**Table 1:** Main controlled clinical studies investigating the effect of different loop diuretic administration and modalities carried out during the last 50 years.

First Author, Publication Year, Study Type	Loop Diuretic Type	Patient Characteristics (n), NYHA Class Age (Years) Sex (M/F)	Administration Modality	Main Endpoints and Findings
Lahav, 1992 [36] prospective, randomized, cross-over, intention to treat	Furosemide	9 III–IV 74 (68–80) 5/4	Continuous furosemide: 30–40 mg of loading dose IV followed by 2.5–3.3 mg/h (60–80 mg/day × 48 h; total dose: 90–120 mg/day) Bolus: 30–40 mg every 8 h × 48 h (total dose: 90–120 mg/day)	Major natriuresis and diuresis in continuous infusion group No significant differences in side effects between two groups
Bagatin, 1998 [37] randomized cross-over, single-blind; intention to treat	Furosemide	12 70.9 ± 6.9 5/7	Continuous: 40 mg IV in 125 cc of 5% dextrose in 1 h × 2 doses Bolus: 40 mg IV × 2 doses	Greater natriuresis and 24 h urinary output in continuous infusion LD vs. bolus LD group
Dormanns, 1996 [38] randomized, cross-over, intention to treat	Furosemide	20 III–IV 71 (51–89) 13/7	Continuous furosemide: 20% of the total dose as loading dose; then, infusion of 10% of the total dose in 8 h Bolus: 1 dose injected in 5 min of a mean of 690 mg (250–2000 mg)	Mean daily urinary volume and sodium excretion were significantly higher after treatment with continuous infusion than with bolus injection
Kramer, 1996 [39] open label, randomized cross-over, intention to treat	Torasemide	8 II–III 44–65 7/1	Continuous torasemide: 25 mg as loading dose, followed by 75 mg in 24 h (3.125 mg/h) Bolus: 100 mg	Continuous vs. bolus torsemide administration resulted in greater 24 h diuresis and natriuresis
Aaser, 1997 [40] prospective randomized cross-over, intention to treat;	Furosemide	8 54 ± 3 6/2	Continuous furosemide: 145 ± 80 mg in 100cc 5% dextrose (range 80–320 mg) × 24 h Bolus: 145 ± 80 mg (range 80–320 mg) given morning (8 am) and afternoon (3 pm)	Bolus LD administration in HF is equally effective as continuous intravenous, which may be related to maximal neurohormonal activation which could not be further activated by bolus administration
Schuller, 1997 [41] prospective, randomized, comparative, intention to treat; method of blinding: not stated	Furosemide	33 18–85 30/3	Furosemide 40 mg then continuous 250 mg in 250 cc of 5% dextrose started at 0.1 mg/kg/h, increased until hourly urine output ≥ 1 cc/kg Bolus: Repeat or double previous dose increased until a net hourly urine output ≥ 1 mg/kg	A tailored patient strategy of diuretic treatment in intensive care is safe and effective
Wu, 2014 [42] randomized single-blind cross-over, intention to treat	Furosemide	20 III–IV 35–75 9/11	Continuous furosemide: 40 mg in 116 cc saline × four h, 2 × day Bolus: 40 mg × three minutes, twice a day	In the treatment of refractory edema in patients with congestive heart failure, continuous intravenous infusion of furosemide is superior to the conventional intermittent bolus injection, especially if it is administered at the very beginning of the hospital treatment,
TORIC STUDY, 2002 [28] open-label prospective cohort	Torasemide	1377 II–III	Oral	The study documented torsemide safety and efficacy in HF treatment and suggested a lower mortality rate in comparison with furosemide
Licata, 2003 [43] randomized, single-blind, intention to treat	Furosemide	107 IV 65–90 39/21	Continuous furosemide: hypertonic saline solution (150 cc of 1.4–4.6% sodium chloride) with 500–1000 mg of furosemide twice a day in 30 min Bolus: 500–1000 mg twice a day without hypertonic saline solution Treatment duration: 6–12 days	Urine output at 24 h Length of hospitalization All-cause mortality, cardiac mortality
DOSE (diuretic optimization strategies evaluation), 2011 [12], prospective-double-blind, randomized trial	Furosemide	308	Continuous infusion and low dose versus double bolus and high dose	The combination of high-dose furosemide and small-volume hypertonic saline solution (HSS) infusion is effective and well tolerated, improves quality of life, and may delay more aggressive HF treatments
DIUR- HF, 2019 [45] single-center, retrospective, observational study	Furosemide	247	Intravenous continuous versus intermittent infusion	Cardiovascular rehospitalization rate was higher in high dose furosemide group (>125 mg median dose) vs. low-dose group (<125 mg median dose)
Frea, 2020 [10] single-center, double-blind, double-dummy, randomized	Furosemide	80	Bolus intermittent vs. continuous infusion	Among patients with acute decompensation of ACHF and high risk of diuretic resistance, continuous infusion of intravenous furosemide was associated with better decongestion

**Table 2:** Different loop diuretic administration timeframes and findings with the use of loop diuretics in chronic heart failure.

Diuretic		Site of Action	Starting Dose	Usual Dose	Half-Life	Onset	Oral Bioavailability
Loop diuretics	Furosemide	Ascending loop of Henle	20–40 mg	40–240 mg	1.5–3.0 h	0.5–1	10–100%
	Bumetanide	Ascending loop of Henle	0.5–1 mg	1–5 mg	1–1.5 h	0.5–1 h	80–100%
	Torasemide	Ascending loop of Henle	5–10 mg	10–20 mg	3–6 h	0.5–1 h	80–100%
Thiazides/thiazide-like diuretics	Bendroflumethiazide	Early distal convoluted tubule	2.5 mg	2.5–10 mg	6–12 h	1–2.5 h	100%
	Hydrochlorothiazide	Early distal convoluted tubule	25 mg	12.5–100 mg	6–15 h	1–2.5 h	65–75%
	Metolazone	Early distal convoluted tubule	2.5 mg	2.5–10 mg	6–20 h	1–2.5 h	60–65%
Non-thiazide sulfonamide	Indapamide	Early distal convoluted tubule	2.5 mg	2.5–5 mg	14–18 h	1–2.5 h	100%
How to use	Monitor renal function and electrolytes; Low starting dose but targeting an effective dose to achieve positive balance of diuresis in parallel with a reduction in body weight by 0.75–1.0 kg per day; Dose adjustment according to patient congestion, blood pressure, and renal function. It is important to use the minimum dose necessary to maintain euvolemia (defined as the ‘dry weight’ of the patients); Re-assess blood chemistry at least 12 weeks after the first dose and after any dose increase (urea/BUN, creatinine); Instruct the patients to modify their own diuretic dose according to specific needs (based on symptoms, signs, and weight changes); When available, the use of specialist HF nurses is helpful in patient education, follow-up (either in person or by telephone), biochemical monitoring, and diuretic dose adjustment.						
Problem solving	Low blood pressure, asymptomatic		Reduce diuretic dose if no symptoms or signs of congestion are detected.				
	Symptomatic hypotension		Dizziness/light-headedness: if no symptoms or signs of congestion, reduce the dose; Evaluate other drugs (e.g., nitrates, vasodilators, calcium channel blockers).				
	Hypokalemia/hypomagnesemia		Increase ACE-I/ARB dose; Add MRA, K and/or Mg supplements.				
	Hyponatremia (<135 mmol/L)		Evaluate patient’s volemic condition; Volume depleted: Stop/reduce thiazide and loop diuretics; Volume overloaded: consider fluid restriction, increasing dose of loop diuretic; AVP antagonist, IV inotropic support; ultrafiltration.				
